# Covert eye-tracking: an innovative method to investigate compliance with instructions

**DOI:** 10.1007/s00426-020-01451-9

**Published:** 2020-12-26

**Authors:** Anine Riege, Amélie Gourdon-Kanhukamwe, Gaëlle Vallée-Tourangeau

**Affiliations:** 1 Department of Management, Kingston Business School, Kingston University, Kingston Hill Campus, Kingston Hill, Kingston upon Thames, KT2 7LB UK; 2 Department of Psychology, University of Oslo, Oslo, Norway; 3 Institute for Globally Distributed Open Research and Education (IGDORE), London, UK

## Abstract

The present study introduces a covert eye-tracking procedure as an innovative approach to investigate the adequacy of research paradigms used in psychology. In light of the ongoing debate regarding ego depletion, the frequently used “attention-control video task” was chosen to illustrate the method. Most participants did not guess that their eyes had been monitored, but some participants had to be excluded due to poor tracking ratio. The eye-tracking data revealed that the attention-control instructions had a significant impact on the number of fixations, revisits, fixation durations, and proportion of long fixation durations on the AOIs (all BF_10_ > 18.2). However, number of fixations and proportions of long fixation durations did not mediate cognitive performance. The results illustrate the promise of covert eye-tracking methodology to assess task compliance, as well as adding to the current discussion regarding whether the difficulties of replicating “ego depletion” may be in part due to poor task compliance in the video task.

## Introduction

Psychological science has for the last decade been engaged in a replication crisis, prompting an increased focus on combatting questionable research practices and attempts to replicate previous results (e.g., the Open Science Collaboration) (for a review see Shrout & Rodgers, 2018). One of the important issues that has received some focus in this debate is the adequacy of the paradigms used in psychological research. If tasks and instructions fail to manipulate the psychological construct under investigation (and only that construct), findings may vary considerably. The present research introduces a novel method of examining the appropriateness of tasks and their instructions by using eye-tracking as a covert measure to evaluate the degree to which participants comply with the task instructions.

Eye movements are seen as a valid measure of attention and cognitive effort (Glaholt & Reingold, 2011; Russo, 2011), and provide information about participants’ acquisition of information, attention and natural shifts in attention through fixations and saccades (Schulte-Mecklenbeck, et al., 2011a, 2011b). Monitoring participants’ fixations within a (pre)defined area of interest (AOI) provides objective information about what participants are looking at and the direction of fixations can indicate search strategies within a display of information, as well as repeated inspections (revisits) of the same material. In addition, the length of each fixation has been used as an indirect measure of cognitive effort, with longer fixations being less common and reflecting a heavier cognitive load than short fixations (Findlay & Kapoula, 1992; Horstmann et al., 2009). As such, eye-tracking methodology provides information about several different psychological processes that are otherwise difficult to access.

To illustrate the use of covert eye-tracking, we used the sequential task paradigm, which is the most common experimental design used for investigating self-control (Carter, et al., 2015). In this paradigm, participants in the experimental condition are required to engage in an initial task requiring use of self-control, such as controlling one’s attention to a specific part of the screen while watching a video. A second, unrelated task, also taxing self-control, is then administered. Worse performance in the second task is taken to be an indicator of the ego depletion effect.

Ego depletion has been a much-debated effect, having failed to replicate in several large-scale replication attempts (Hagger, et al., 2016; Lurquin, et al., 2016) and showing weak effect sizes in meta-analyses (Carter, et al., 2015). There are several reasons why an ego depletion effect may be difficult to observe (Blazquez, et al., 2017; Cunningham & Baumeister, 2016), such as the existence of ego depletion effect itself or, if it exists, whether self-control as a depletable resource is an appropriate theoretical framework to account for the ego depletion effect (e.g., Baumeister, et al., 1998, 2007; Inzlicht & Schmeichel, 2012; Kotabe & Hofmann, 2016). Yet, another possible important issue is methodological, where the adequacy of the tasks used to elicit ego depletion has been questioned (Lee, et al., 2016). Different meta-analyses have suggested that not all manipulations or dependent variables used in the literature may work (Dang, 2018; Lurquin & Miyake, 2017). The attention-control video, for example, has been shown in a replication paper (Lurquin, et al., 2016) and a meta-analysis (Dang, 2018) to potentially be a weak task to manipulate depletion of self-control. However, less is known about why the attention-control video might be a weak task—it might not require self-control or participants might not follow the instructions.

In the attention-control video task, participants watch a six-minute silent video of a woman being interviewed by an offscreen interviewer. During the video, 36 common one-syllable words (e.g., play) appear at the bottom of the screen for 10-s each. The ego depletion manipulation supposedly involves taxing self-control resources of one group but not the other, through manipulating the video-watching instructions. In the taxing condition, participants are instructed to control their visual attention and only concentrate on the woman. In the untaxing condition, participants complete the same task without such instructions and without being informed about the presence of the words. Thus, participants in the taxing group are assumed to use self-control resources to avoid looking at the words and, thus, are predicted to become depleted in self-control compared to those in the untaxing group (Lee, et al., 2016). In other words, the changing words potentially draw participants’ attention in a bottom-up way. However, participants in the taxing condition must override such bottom-up demands, presumably using top-down control because they are instructed to not look at the words, which is taxing.

As such, participants’ compliance with the instructions is a crucial pre-condition for this self-control manipulation to impact performance. If participants in the taxing condition look at the words, they may be less depleted than assumed; conversely, if participants in the untaxing condition either try to avoid the words or try to commit the words to memory they may be more depleted than they are assumed to be. In other words, participants who fail to comply with instructions in either condition may diminish the effectiveness of the self-control manipulation intended to induce the ego depletion effect. Self-report data suggest that participants in the taxing condition may not fully comply with task instructions as they do remember some words (Henderson, et al., 2017; Lurquin, et al., 2016); however, such self-report data only provide a proxy measure for task compliance. What is needed is a more objective measure of behavior during the task.

**Fig. 1:**
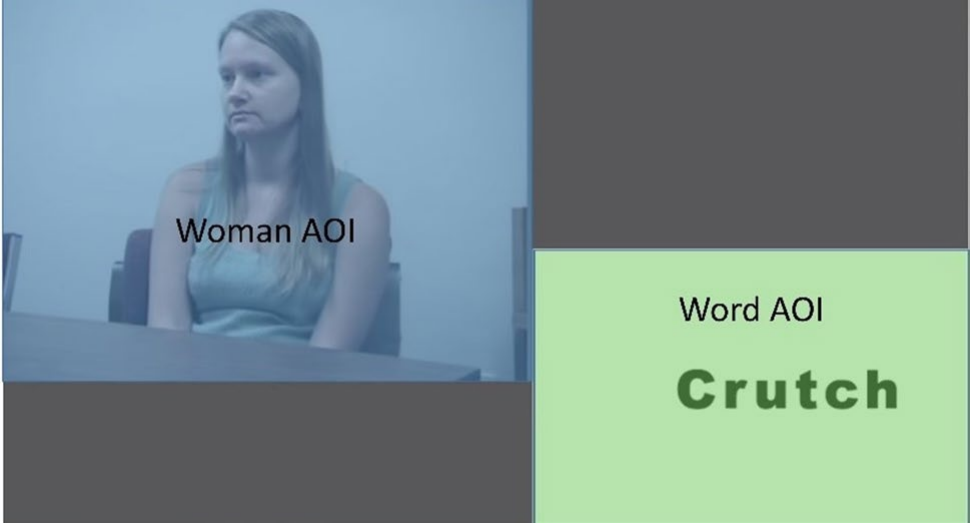
The AOIs used to extract data. The blue area (top, left) is the woman-AOI and the green area (bottom, right) is the word-AOI

The attention-control video can be viewed as a task where the words appearing at bottom right of the screen draw the gaze. Such bottom-up effects can be hard to ignore and requires exerting control over one’s eye movements to avoid looking. Eye movements such as fixations, revisits, and fixation durations provide such objective measures of the degree to which participants comply with task instructions and should differ based on the self-control condition participants are randomly allocated to (taxing vs. untaxing). Using (pre)defined AOIs (see Fig. 1) provides objective information about how often participants are looking at either the woman or the words (number of fixations) and how many repeated inspections (revisits) of an AOI do not follow each other in time. In other words, participants in the taxing condition who fully comply with the task instructions should have no fixations and no revisits to the Word-AOI because they do not look at the words. However, the present paradigm allows for a more fine-grained understanding of participants’ compliance as the combination of the number of fixations and revisits gives objective data of the extent of compliance.

In the present paradigm, it is also possible that the task is so easy that participants manage to not to look at the words at all. The length of each single fixation (measured in milliseconds) is often used as an indirect measure of cognitive effort. Fixation durations vary based on the activity at hand, where fixation durations longer than 500 ms often indicate a heavier cognitive load. For example, for participants calculating weighted sums in a city-size task (pre-study), the proportion of long fixation durations (> 500 ms) was 18.5% compared to 1.4% of all fixations for participants processing simple city-size tasks more intuitively (Study 1) (Horstmann, et al., 2009). As such, using self-control resources should require more effort, meaning that participants in the taxing condition should have longer fixation durations on the Woman-AOI compared to participants in the untaxing condition. Fixation durations on the woman should also potentially be longer than those on the words for both conditions because the task is to observe the woman’s body language. Taken together, eye-tracking provides several indices of participants’ attention processes/behavior, making it a useful tool to investigate the adequacy of research paradigms in psychology.

While eye-tracking is a useful method, it is not necessarily practical to include eye-tracking in every research study. The purpose of the present method is rather to understand whether participants are following instructions when they are not observed, which is how experiments are typically run. However, the Hawthorne effect suggests that knowing one’s being observed might change participants’ behavior (McCambridge, et al., 2014). As eye movements can be controlled and we wanted to investigate if participants followed the instructions not to look at the words, it was important for participants to be unaware that their eye movements are being tracked during the task completion. Overt eye-tracking would make it difficult to extrapolate to attention-control paradigms in general, thus we used covert eye-tracking. Next, we report an experiment showcasing a new approach to assess task compliance, using covert recording of eye movements.

The main aim of the paper was, thus, to shed light on whether the attention-control task failed to produce an ego depletion effect because of non-compliance with task instructions, using covert eye-tracking. As a second aim, we also tested whether the impact of the self-control manipulation on performance was moderated by the extent to which participants complied with the video-viewing task instructions. We replicated the method and materials from Schmeichel et al. (2003, Experiment 1) as closely as possible, adding eye-tracking to investigate whether participants actually engage in attentional control behavior while completing the video-viewing task by covertly monitoring gaze patterns.

### Hypotheses

Following Schmeichel, et al. (2003), we used different instructions to the attention-control video as the independent variable and tasks from the analytical section of the Graduate Record Exam (GRE) as the dependent variable. H1a, thus, tests whether ego depletion from the attention-control video task occurred, as measured by Schmeichel, et al. (2003), where the main measure of performance is the number of correct responses, the proportion of correct responses indicates overall accuracy, and the number of attempted tasks is a measure of working speed and effort.

H1b and H1c test whether ego depletion occurred as measured by the attentional control exerted by participants.^1^ Hypotheses H2a and H2b test whether participants followed the instructions of the attention-control video task (intended to deplete resources), via the number of fixations in the Word-AOI (H2a) and number of revisits to the Word-AOI (H2b). Finally, H3a tests the effort exerted by participants by comparing the proportion of long fixation durations (> 500 ms) in the Woman-AOI. We, therefore, set out to either support or refute the following pre-registered hypotheses:

H1a: participants in the taxing condition will perform worse on the three performance measures of the GRE, compared to the untaxing condition.

H1b: performance on the GRE will be mediated by the number of fixations in the Word-AOI.

H1c: performance on the GRE will be mediated by effort (measured as proportion of long fixation durations).

H2a: participants in the taxing condition will have fewer fixations in the Word-AOI compared to the untaxing condition.

H2b: participants in the taxing condition will have fewer revisits to the Word-AOI compared to the untaxing condition.

H3a: participants in the taxing condition will have longer fixation durations in the Woman-AOI compared to the untaxing condition.

Prior to data collection, we pre-registered the hypotheses, method and planned analyses on the Open Science Framework https://osf.io/puh6t/. We report all pre-registered analyses below, except the exploratory pupillometry analyses.^2^

## Materials and method

### Sampling plan

A sequential Bayesian analysis (Schonbrodt, et al., 2017) using the JASP default Cauchy prior (*r* = 0.707) was conducted on previously collected data (Henderson, et al., 2017) to determine the stopping rule. A Bayes factor of ~ 5 was reached after 70 participants. We pre-registered to stop recruiting either when a Bayesian *t*-test on the dependent variable (GRE) indicated that the data were five times more likely under H1 than H0 (i.e., BF_10_ = 5) or vice versa (i.e., BF_01_ = 5), or at 80 participants. Due to more sign-ups than anticipated, data collection was stopped at 85 participants. The experimental procedures were approved by the University of Kingston’s Research Ethics Committee. The dataset generated and analyzed in the current study is available in the OSF repository, at https://osf.io/chnrm/.

### Participants

Eighty-five participants were recruited, each receiving a £10 Amazon voucher for their participation. A total of 15 participants were excluded, six for pre-registered reasons (4 participants were aware of eye-tracking, 1 participant had problems with eyesight, and 1 chose to retract data). Two unregistered exclusion criteria were added: receiving incorrect instructions (2 participants), and tracking ratio < 70% (7 participants; although we had pre-registered poor quality eye-tracking data, we did not register to follow recommendation of Amso, et al., 2014). Tracking ratio is the proportion of time that the eye-tracker records the gaze during the task, and although participants were given instructions to not look away from the screen, the covert eye-tracking meant they did not have the same awareness and instruction ordinarily given to increase tracking ratio. The mean tracking ratio in the excluded group was 45.7% (SD = 21.9), compared to 89.9% (SD  = 6.6) for the included participants. The accuracy of the eye-tracking data was on average *X*[°] = 0.39 (SD = 0.25) and *Y*[°] = 0.59 (SD = 0.57). Two participants had rather large *Y*-deviations (3.3 and 3.4), but manual inspection of the participants’ gaze videos revealed that the size and the positioning of the AOIs (see Fig. 1) prevents the gaze being recorded in the incorrect AOI.

Thus, 70 participants (52.9% women, mean age = 24.2 years, SD = 6.7) with normal or corrected-to-normal vision and fluent in English were included in the analyses.

### Design and materials

Participants were randomly allocated to either a taxing or untaxing self-control condition, differing only in the instructions given for the video-viewing task. All tasks were completed in a single testing session, lasting ~45 min.

Participants were first given an information sheet and consent form, and demographic questionnaire. Next, they were positioned at 65-cm distance to the screen, which was fitted with an SMI RED 250 screen-based mobile eye-tracker, and told they were doing a brief concentration task, adapted from the SAM lab’s instructions (B. Lassetter, personal communication) and which served as a covert procedure for calibrating the eye tracker. Participants’ eye movements were recorded at 60 Hz, using a 5-point calibration procedure. Participants were instructed to face their monitor comfortably while the experimenter would measure the distance between their head and the screen. They were told to keep their head still and maintain the distance while they looked at the screen. Participants were then told that a circle would appear, move across the screen, and pause once in a while. They were instructed to count in their heads the number of times the circle paused and to report this number to the experimenter at the end of the concentration task. By following the circle with their eyes, unbeknownst to them, participants were actually providing the data required to calibrate the eye-tracker. If the calibration was noisy (that is, accuracy > 1°), the experimenter would repeat the procedure. If the calibration failed (i.e., it was not possible to get an accurate validation) after five attempts, the participant was thanked, debriefed, and dismissed (verbatim instructions to participants can be found on OSF, in the protocol document available at https://osf.io/xxaz6/).

After the best possible validation was obtained (preferably < 1°), the instructions to the video task were given. The video was displayed on a 22-in. monitor with a resolution of 1680 × 1050 pixels. Participants viewed the 6-min video while the experimenter waited outside. The instructions to the video-viewing task for participants in the taxing group were:

“This experiment investigates how people form impressions of others and how those impressions influence memory. So, I’m going to have you watch a short film clip that shows a woman being interviewed, but I’m going to turn the sound off so that you can only see the woman. Later I’ll have you answer some questions about your impressions of her. Since you won’t be able to hear what she’s saying you’ll have to base your impressions of her on her nonverbal behavior.

So, in addition to the woman being interviewed, you will also see some words on the bottom of the screen. It is very important for the purposes of this experiment that you keep your attention focused only on the woman’s face and do not look down at the words that appear at the bottom of the screen. If you do accidentally look at the words, I want you to re-focus your attention on the woman as quickly as possible.

This task may be kind of difficult because the words take up a decent portion of the screen, but I want you to try hard to ignore these words and focus your attention only on the woman. Later, you’ll have to answer some questions about your impressions of the woman based on her nonverbal behavior. When the clip ends, let me know. Remember, focus only on the woman and try to ignore the words”.

For the participants in the untaxing group, the instructions to the video-viewing task were:

“This experiment investigates how people form impressions of others and how those impressions influence memory. So, I’m going to have you watch a short film clip that shows a woman being interviewed, but I’m going to turn the sound off so that you can only see the woman. Later I’ll have you answer some questions about your impressions of her. Since you won’t be able to hear what she’s saying you’ll have to base your impression of her on her nonverbal behavior.

When the interview clip starts, I want you to watch it just as if you were sitting at home watching TV, even though the sound will be off. I don’t want you to worry about try real hard to form an impression or anything. Just watch the clip and when it ends, let me know”.

After the participants had watched the attention control video, the PANAS mood scale (Watson, et al., 1988) was administrated for replication purposes, but these data were not analyzed or reported, as specified in the pre-registration. Participants were given 13 problems from the Analytical section of the Graduate Record Exam (GRE). These tasks are considered to be a measure of general cognitive ability (Kuncel, et al., 2001) that requires active cognitive control and self-regulated thinking (Yang & Johnson-Laird, 2001), for example:

Seven offices in an office building are to be painted. The offices, which are on one side of a hallway, are numbered consecutively, one to seven, from the front of the building to the back. Each office is to be painted one color only according to the following conditions: Two offices must be painted white; two offices must be painted blue; two offices must be painted green; and one office must be painted yellow. The two offices painted green must be next to each other. The two offices painted blue cannot be next to each other. The office painted yellow cannot be next to an office painted white. Office 3 must be painted white. 1) If office 2 is painted green, which of the following offices must also be painted green? (Answer alternatives were: a. 1; b. 3; c. 4; d. 5; e. 6).

After having spent 10 min on the GRE, participants were interrupted by the experimenter. Following Lurquin, et al., (2016), participants filled out a surprise video memory task and a video-viewing scale. The memory task asked participants to state whether or not they remembered seeing each of 36 words (Yes/No), where half were words that had appeared in the video and the others were decoy words. The video-viewing scale is an eight-question self-report measure asking participants to rate questions such as “How difficult was it not to look at the words?” and “How hard did you try to remember the words?” on a scale from 1 (Not at all) to 10 (A lot).

Lastly, participants filled out a post-experiment questionnaire, probing for detection of the eye-tracker. Participants were then debriefed and asked again for consent once the covert eye-tracking had been revealed. All materials can be found on OSF: https://osf.io/xxaz6/.

The size of the Woman-AOI was 845,983 pixels (40.2% of the screen) and the Word-AOI was 492,936 pixels (23.4% of the screen). The AOIs are displayed in Fig. 1.

## Results

The pre-registered hypotheses are labeled, and the other analyses are included for transparency. We used JASP for all Bayesian *t*-tests, using the default Cauchy prior (*r* = 0.707). Following Schmeichel, et al., (2003), we analyzed the performance data from the GRE in three different ways, where the number of correct responses is the main measure of performance, the proportion of correct responses gives an indication of overall accuracy, and the number of attempted tasks is a measure of working speed and effort. As seen in Table 1, the performance data on the GRE were between 3.7 and 4.9 times more likely under the null hypothesis (*H*_0_) rather than under the alternative hypothesis (H1a), providing moderate evidence that participants’ performance on the GRE did not differ as a function of self-control condition.

**Table 1:** Mean (SD) GRE scores for participants in each group, and the Bayes Factor for *H*_0_ for an independent *t*-test and a traditional students *t*-test (hypothesis H1a)

	Self-control condition								
	Taxing		Untaxing						
	*M*	SD	*M*	SD	BF_0+_	*t*(68)	*p*	Cohen’s *d* (95% CI)	
GRE_correct_	2.6	(1.4)	2.8	(1.5)	3.68	0.47	0.637	0.113 (− 0.36, 0.58)	
GRE_proportion_	0.5	(0.2)	0.5	(0.3)	4.47	0.26	0.798	0.062 (− 0.44, 0.50)	
GRE_attempted_	6.3	(2.9)	6.4	(2.91)	4.92	0.14	0.886	0.034 (− 0.41, 0.53)	

GRE_correct_ = number of GRE questions participants got correct; GRE_proportion_ = proportion of GRE questions participants got correct; GRE_attempted_ = number of GRE questions participants attempted

To investigate whether the degree of task compliance mediates the effect of the self-control manipulation on GRE performance, we ran three mediation analyses using PROCESS version 3.5, Model 4 (Hayes, 2018) in SPSS. The first mediation analysis (hypothesis H1b) looked at the effect of condition (taxing vs. untaxing) on the GRE_correct_ score, mediated by the number of fixations in the Word-AOI. Condition predicted the number of fixations in the Word-AOI, *t*(68) = − 8.25, *p* < 0.001, but there was no significant indirect effect of condition through number of fixations on overall GRE performance, ab = − 0.33, 95% CI [− 0.74, 0.21]. In other words, no mediation occurred. The second model (hypothesis H1c) looked at the effect of condition on the GRE_correct_ score, mediated by proportion of long fixation durations (> 500 ms). Condition predicted the proportion of long fixation durations, *t*(68) = 6.09, *p* < 0.001, but there was no significant indirect effect of condition on overall GRE performance through proportions of long fixation durations, ab = − 0.17, 95% CI [- 0.43, 0.06] indicating no mediation occurred. The pre-registration also included checking if self-reported effort (see Q3 in Table 3) mediated the effect of condition on the GRE_correct_ score (also hypothesis H1c). Condition did not predict self-reported effort, *t*(68) = 0.36, *p* = 0.718, and there was no significant indirect effect of condition through self-reported effort on overall GRE performance, ab = − 0.01, 95% CI [− 0.12, 0.05], meaning no mediation occurred.

We tested hypotheses H2a, H2b, and H3a to investigate if participants followed the instructions of the attention-control video task comparing the number of fixations, revisits, and fixation durations between the two conditions, respectively. As seen in Table 2, participants in the taxing condition had substantially fewer fixations and revisits in the Word-AOI, with Bayes factors well above the criteria for “extreme evidence” (Lee & Wagenmakers, 2013, adjusted from Jeffreys, 1961) for hypotheses H2a and H2b. Further inspections of the data revealed that 32.4% of participants in the taxing condition had no fixations in the Word-AOI, meaning that they completely adhered to the instructions and never looked at the words. Less than 20% of the participants in the taxing condition had more than 10 fixations in the Word-AOI. Revisits are repeated inspections of an AOI that do not follow each other in time, while fixations may or may not follow each other in time. Though they are closely related to fixations, revisits show how many times participants returned to the Word-AOI. Only six participants (16.2%) in the taxing condition had 10 or more revisits to the Word-AOI, where only two had 25 and 30 revisits. In comparison, 84.8% of participants in the untaxing condition had more than 25 revisits, where 9.1% had more than 100 revisits.

**Table 2:** Mean (SD) fixations, revisits and fixation durations for the two AOIs by condition, together with results for independent *t*-tests where the Bayes Factor is in favor of H1 (group 1 ≠ group 2)

	Self-control condition								
	Taxing		Untaxing						
	*M*	SD	*M*	SD	BF_10_	*t*(68)	*p*	Cohen’s *d* (95% CI)	
Fixations woman	238.1	(109.7)	315.9	(88.8)	18.2	3.2	0.002	0.78 (0.29, 1.26)	
Fixations words (hypothesis H2a)	6.5	(10.2)	103.7	(70.9)	8.0e + 8	8.3	< 0.001	1.98 (1.40, 2.55)	
Revisits woman	8.4	(10.1)	66.8	(28.6)	4.6e + 14	11.7	< 0.001	2.79 (2.12, 3.45)	
Revisits words (hypothesis H2b)	4.1	(6.9)	59.4	(30.1)	2.5e + 13	10.9	< 0.001	2.61 (1.96, 3.24)	
Fixation durations woman (ms) (hypothesis H3a)	1824.7	(1270.5)	904.9	(381.9)	147.9	− 4.0	< 0.001	− 0.96 (− 1.45, − 0.46)	
Fixation durations words (ms)	152.0	(140.8)	348.4	(146.4)	45,504.1	5.7	< 0.001	1.37 (0.84, 1.89)	
Proportion long fixation durations	0.59	(0.14)	0.40	(0.11)	184,649.6	− 6.1	< 0.001	− 1.46 (− 1.98, − 0.93)	

Participants’ fixation durations also varied between the two conditions (see Table 2). Hypothesis 3a also had a Bayes Factor (BF_10_) above 100 denoting “extreme evidence” in favor of research hypothesis H3a. Fixation durations in the Word-AOI were notably shorter and in the opposite direction, indicating that participants in the taxing condition who did look at the Word-AOI had more fleeting glances compared to those in the untaxing condition. The proportion of participants’ fixation durations which were above 500 ms was also significantly higher in the taxing condition.

Finally, the self-report measures for the video-viewing task and the memory test are reported in Table 3. The calculation of the total memory score follows Lurquin & Miyake’s (2017) signal detection analysis calculating *d*-prime (*d*’). Specifically, *d*’ represents the ability to distinguish between decoy words and displayed words, by considering both the hit rate (words that are correctly labeled as have seen/have not seen) and the false alarm rate (decoy words that are incorrectly remembered as seen). As such, *d*’ corrects for participants’ guessing on all the words. For example, if the measure of the memory task was the proportion of words correctly remembered, and a participant responded “Yes” to all the words, it would seem like they had looked at all the words unless the false alarms are also taken into account.

**Table 3:** Mean (SD) scores for the memory task and the video-viewing task, and the results of independent *t*-tests

	Self-control condition							
	Taxing		Untaxing					
	*M*	SD	*M*	SD	*t*(68)	*p*	Cohen’s *d* (95% CI)	
Q1. How difficult was it not to look at the words?	4.2	(2.7)	6.1	(2.2)	− 3.12	0.003	0.75 (0.26, 1.23)	
Q2. How difficult was it to form an impression of the interviewee?	5.2	(2.2)	5.3	(2.1)	− 0.18	0.860	0.04 ( − 0.43, 0.51)	
Q3. How much effort did you put into the task?	7.6	(2.2)	7.4	(1.7)	0.36	0.719	− 0.09 (− 0.56, 0.38)	
Q4. Did you think we would ask about the words later?	5.0	(3.4)	8.6	(2.1)	− 5.25	< 0.001	1.26 (0.74, 1.77)	
Q5. How hard did you try to remember the words?	2.9	(2.4)	6.8	(2.4)	− 6.83	< 0.001	1.63 (1.09, 2.17)	
Q6. How hard did you try to ignore the words?	7.1	(2.9)	4.3	(2.5)	4.29	< 0.001	− 1.03 (− 1.52, − 0.52)	
Q7. How important was it for you to see the words?	3.0	(2.3)	5.5	(2.5)	− 4.55	< 0.001	1.09 (0.58, 1.59)	
Q8. How important was it to you to follow the instructions?	8.1	(2.3)	5.5	(2.4)	4.61	< 0.001	− 1.10 (− 1.61, − 0.60)	
Memory of words in the video	3.5	(3.5)	12.3	(4.2)	− 9.64	< 0.001	2.31 (1.69, 2.91)	
Memory of decoy words (i.e., words that did not appear in the video)	1.3	(1.8)	2.0	(1.5)	− 1.70	0.093	0.41 (− 0.07, 0.88)	
*d*’ for memory of words	0.35	(0.4)	1.4	(0.6)	− 8.12	< 0.001	− 1.97 (1.40, 2.55)	

The video-viewing task and the memory test revealed some interesting differences. For example, the questions probing for effortful processing of the words (Questions 4, 5, and the memory scores in Table 3) show that participants in the untaxing condition not only thought it more likely that they would be asked about the words and tried to remember the words, they were also better able to distinguish between words shown in the video and decoy words (as indicated by *d*’). This suggests that several participants in the untaxing condition may have exerted effort by trying to commit the words to memory. Participants in the untaxing condition also reported it as more difficult to not look at the words (Q1, Table 3). However, this might be because the instructions for this group does not mention the words at all, making participants in the untaxing condition unsure about the presence of the words. For the question asking about how hard participants tried to ignore the words (Q6), participants in the taxing condition reported significantly higher ratings, which is in line with what one would expect from the instructions. Participants reported experiencing similar difficulty in forming an impression of the woman (Q2) and putting similar effort into the task (Q3).

Taken together, our results indicate that participants do attempt to follow the instructions, yet both groups perform equally on the GRE, indicating no ego depletion effect. However, the self-report data suggest that participants in the untaxing condition may have tried to commit the words to memory, and to investigate whether participants in the untaxing condition could also be using attentional resources we decided to do two exploratory analyses of the proportion of long fixation durations in the Word-AOI. An independent *t-*test (excluding participants with no fixations in the Word-AOI) showed that participants in the untaxing condition had a significantly higher proportion of long fixation durations when looking at the Word-AOI (*M*_taxing_ = 0.042 vs. *M*_untaxing_ = 0.132, *t*(56) = 3.46, *p* = 0.001, BF_10_ = 30.3). The second exploratory analysis looking at the correlations between the proportion of long fixation durations and the questions in the video-viewing task (Table 3) showed four correlations with Bayes factors indicating strong (10–30) and moderate evidence (3–10) (Lee & Wagenmakers, 2013, adjusted from Jeffreys, 1961). More specifically, the questions asking whether participants attempted to memorize the words all showed positive correlations with proportion of long fixation durations, indicating that participants with more long fixation durations gave higher ratings to the questions. Question 4 (Did you think we would ask about the words later?) had a correlation of *r*(58) = 0.37, BF_10_ = 7.9; Question 5 (How hard did you try to remember the words?) had a correlation of *r*(58) = 0.35, BF_10_ = 5.4; and Question 7 (How important was it for you to see the words?) had a correlation of *r*(58) = 0.38, BF_10_ = 12.0. Conversely, the question asking participants how important it was to follow the instructions showed a negative correlation with proportion of long fixation durations (Q8. How important was it to you to follow the instructions?) had a correlation of *r*(58) = − 0.37, BF_10_ = 8.4.

## Discussion

The present study introduces covert eye-tracking as a method to investigate the adequacy of research paradigms used in psychology. The frequently used “attention-control video task” was chosen to illustrate the method. Most participants did not suspect that their eyes had been monitored, indicating that covertly tracking participants’ eye movements is an unobtrusive way to obtain reliable behavioral data on participants’ compliance with visual attention instructions. Moreover, this method ensures that participants are not controlling their eye movements as they might be with overt eye-tracking. Further, the eye-tracking data provided an opportunity to investigate whether individual differences in adherence affects the outcome measure at hand.

One possible limitation of this approach, however, is that it might have resulted in participants having a poorer tracking ratio than normal. A poor tracking ratio means that a lot of data are missing, although some data loss is normal (Holmqvist, 2011). Participants blink, they might have eye lids or eye lashes that obscure the pupil or corneal reflection (which are both used to track the gaze position), and they may on occasion look away from the screen. As it is more difficult to control for such situations when eye-tracking is done covertly, this could be a disadvantage with covert eye-tracking. Future operationalizations of the covert eye-tracking method for attentional tasks may consider including instructions to invite participants to limit blinking and keep their eyes properly open.

We were also able to illustrate the usefulness of the covert eye-tracking methodology to disentangle methodological biases from theoretical and statistical biases to explain the absence of an effect. Using the ego depletion as an example, this study illustrates that the absence of an ego depletion effect, following the attention-control video task, seems unlikely to be attributed to participants’ failing to comply with the “ego-depleting” instructions. On the contrary, participants in the taxing condition had few fixations and revisits to the Word-AOI, indicating that they mostly followed the instructions. Further, the length of the few fixations they had on the words were only (on average) 152 ms long, which indicates quite short glances (the recommendation for what constitutes a fixation is normally fixation durations between 100 and 200 ms, see Manor & Gordon, 2003).

Understanding why and how an experimental task works is important to move research on self-control forward. We reasoned that if participants in the taxing experimental condition were not exerting effort to look away from the words, they would not be ego depleted. Our eye-tracking measures revealed that participants in the taxing condition had significantly fewer fixations and revisits to the Word-AOI compared to the untaxing condition, and many participants had none. These results show that the video-viewing task instructions do have an impact on self-control behavior (i.e., the eye-tracking patterns clearly differ in the taxing condition, compared to the untaxing condition), yet this impact does not result in a change of performance in the outcome task. In fact, the data provided moderate evidence against an ego depletion effect on the GRE performance. As anticipated, participants varied in their degree of compliance, but compliance did not moderate the effect of the self-control manipulation on the GRE performance. As such, the lack of evidence for an ego depletion effect cannot be attributed to a poor manipulation of participants’ self-control in the video task.

The results also showed that participants in the taxing condition had longer fixation durations when looking at the Woman-AOI. Long fixation durations have been shown to indicate cognitive effort which is in line with the purpose of the task’s manipulation. However, given the results of the mediation analysis, long fixation durations may not only indicate cognitive effort in the present experiment, as studies on eye movements while driving in monotonous landscapes (i.e., not effortful environments) also find long fixation durations (Chapman & Underwood, 1998).

The present study aimed to investigate not only if participants in the taxing condition follow instructions, but also if participants in the untaxing condition might either try to avoid the words or try to commit the words to memory leaving them more depleted than expected. There was little evidence that participants in the untaxing condition tried to avoid looking at the words. As seen in Table 2, there is a large difference between the two conditions regarding fixations in the Word-AOI. A closer inspection of the data revealed that only one participant in the untaxing condition had less than 10 fixations to the Word-AOI and almost half had more than 100 fixations. On the other hand, our self-report data suggest that participants in the untaxing condition may have tried to commit the words to memory as they reported significantly higher responses to the questions probing for effortful processing of the words. Participants in the untaxing condition also remembered far more words and had a higher d-prime score, indicating that participants in the untaxing condition were better able to distinguish between words shown in the video and decoy words. Taken together, several participants in the untaxing condition may have exerted considerable effort committing the words to memory. As seen in our exploratory analyses of the proportion of long fixation durations in the Word-AOI, our results indicate that the problem with the task may not be a lack of compliance in the taxing condition, but participants exerting effort in the untaxing condition by inferring additional requirements. A recent study also proposing to examine the validity of the attention-control video for the ego depletion effect (Englert, et al., 2019) used the attention-control video task as a dependent measure. The results showed that participants in the taxing condition had significantly fewer fixations on the Woman-AOI compared to participants in the untaxing condition indicating that they were depleted from the preceding manipulation. This is a less common use of the task, as it has generally been used as a means to manipulate attentional resources (Carter, et al., 2015), but it indicates the task may indeed be tapping into self-control.

One solution might be to change the wording of the instructions, particularly for the untaxing condition. Recall the instructions for both conditions begin with “This experiment investigates how people form impressions of others and how those impressions influence memory.”, because there is no mention of the words in the instruction for the untaxing condition, some participants may think the mention of memory is related to memorizing the words. This might also be a potential reason for the finding that participants in the untaxing condition reported that it was more difficult to not look at the words (Q1 in Table 3), compared to the taxing condition (where participants received instructions to not look at the words). The question might have been interpreted slightly differently by participants in the untaxing condition as they did not expect to see the words in the video.

One important caveat of the present study is that although we made every effort to replicate the original Schmeichel et al.’s study (2003, Experiment 1), the average GRE performance observed in our study was much lower than that observed by Schmeichel, et al., (2003). This floor effect in the GRE performance may be due to cultural differences between Schmeichel’s US sample and our UK sample, since the GRE is typically used in the US education system where in the UK it is not.

The present work introduced covert eye-tracking as an approach to investigate the adequacy of psychological research paradigms. Very few participants realized that their eyes had been monitored, but some participants had to be excluded due to poor tracking ratio. The results showed that participants were complying with the instructions as the gaze patterns differed substantially between the two conditions. However, number of fixations and proportions of long fixation durations did not mediate participants’ performance on the GRE. As such, lack of participant compliance does not appear to explain why we, and others, have failed to observe an ego depletion effect resulting from the attention-control video task. Beyond research on the ego depletion effect, the covert eye-tracking methodology introduced here has the potential to examine the adequacy of both old and new research paradigms within psychology where instruction compliance is a key methodological constraint.

## Footnotes

1 The wording in H1b and H1c is slightly changed from the pre-registered version (omitting “regardless of condition”) to avoid confusion as the pre-registered analyses included conditions. We found the syntax more important to follow than the wording in the hypotheses.

2 The pre-registration included H3b (During the video-viewing task participants in the depletion condition will have larger mean pupil size, compared to the control condition) as an exploratory analysis. After having pre-registered the study, we discovered a potential confounding issue which is that the luminance in the two AOIs might be different (see Fig. 1), and luminance also affects pupil size (see for example Campbell & Gregory, 1960; Holmqvist, 2011, chapter 11.5; Laeng & Sulutvedt, 2014; Sulutvedt, et al., 2021). Moreover, we expect the luminance in the Word-AOI to be brighter than in the Woman-AOI which means that participants’ pupils might contract (indicating less effort) when they look at the Word-AOI. If this is the case, any observed change in pupil size in the predicted direction could be caused by either a reduced cognitive effort or by the change in luminance (or both). For this reason, we have not looked at the pupil data.
